# Naturally acquired antibody response to *Plasmodium falciparum* describes heterogeneity in transmission on islands in Lake Victoria

**DOI:** 10.1038/s41598-017-09585-4

**Published:** 2017-08-22

**Authors:** Zulkarnain Md Idris, Chim W. Chan, James Kongere, Tom Hall, John Logedi, Jesse Gitaka, Chris Drakeley, Akira Kaneko

**Affiliations:** 1 Department of Parasitology and Medical Entomology, Faculty of Medicine, Universiti Kebangsaan Malaysia Medical Centre, Kuala Lumpur, 56000 Malaysia; 2 Island Malaria Group, Department of Microbiology, Tumor and Cell Biology, Karolinska Institutet, Stockholm, 17177 Sweden; 3 Nagasaki University Nairobi Research Station, NUITM-KEMRI Project, Nairobi, 00202 Kenya; 4 Department of Immunology and Infection, London School of Hygiene & Tropical Medicine, London, WC1E 7HT UK; 5 National Malaria Control Programme, Ministry of Public Health and Sanitation, Nairobi, 00100 Kenya; 6 Department of Clinical Medicine, Mount Kenya University, Thika, 01000 Kenya; 7 Department of Parasitology and Research Centre for Infectious Disease Sciences, Graduate School of Medicine, Osaka City University, Osaka, 558-8585 Japan; 8 Institute of Tropical Medicine, Nagasaki University, Nagasaki, 852-8102 Japan

## Abstract

As markers of exposure anti-malaria antibody responses can help characterise heterogeneity in malaria transmission. In the present study antibody responses to *Plasmodium falciparum* AMA-1, MSP-1_19_ and CSP were measured with the aim to describe transmission patterns in meso-endemic settings in Lake Victoria. Two cross-sectional surveys were conducted in Lake Victoria in January and August 2012. The study area comprised of three settings: mainland (Ungoye), large island (Mfangano) and small islands (Takawiri, Kibuogi, Ngodhe). Individuals provided a finger-blood sample to assess malaria infection by microscopy and PCR. Antibody response to *P*. *falciparum* was determined in 4,112 individuals by ELISA using eluted dried blood from filter paper. The overall seroprevalence was 64.0% for AMA-1, 39.5% for MSP-1_19_, and 12.9% for CSP. Between settings, seroprevalences for merozoite antigens were similar between Ungoye and Mfangano, but higher when compared to the small islands. For AMA-1, the seroconversion rates (SCRs) ranged from 0.121 (Ngodhe) to 0.202 (Ungoye), and were strongly correlated to parasite prevalence. We observed heterogeneity in serological indices across study sites in Lake Victoria. These data suggest that AMA-1 and MSP-1_19_ sero-epidemiological analysis may provide further evidence in assessing variation in malaria exposure and evaluating malaria control efforts in high endemic area.

## Introduction

In sub-Saharan Africa, malaria remains one of the leading causes of morbidity and mortality, with 191 million cases and over 390 thousand deaths reported in 2016^1^. Nevertheless, with scaling up of malaria prevention, diagnosis and treatment, the prevalence of *Plasmodium falciparum* infection in many parts of sub-Saharan Africa declined by 50%, and the incidence of clinical disease fell by 40% between 2000 and 2015^2^.

In Kenya, 65% (26 million) of the population live in areas where *P*. *falciparum* parasite rate for the population aged 2–10 years (P*f*PR_2–10_) is below 1%^3^. Despite significant increases in coverage of long-lasting insecticide-treated nets (LLINs) through free-mass distribution campaigns^4^ and the change in first-line treatment to artemisinin-based combination therapy (ACT)^5^, 10.6% (4.3 million) of the population still live in areas with P*f*PR_2–10 ≥ _40%, mainly counties adjacent to Lake Victoria in western Kenya^6^. In addition, increased paediatric malaria hospital admissions in 2009^7^ and high prevalence of malaria infection among school children in 2014^8^ highlight the challenge of effective malaria control in this highly endemic region of Kenya.

Understanding the intensity of malaria transmission in a community is fundamental to the design and evaluation of malaria control and elimination programmes. The most widely used metric of malaria transmission intensity is parasite prevalence, measured through cross-sectional surveys. In recent years, antibody responses to one or more malaria parasite-specific antigens have been explored as alternative means to estimate malaria transmission intensity^9, 10^. As a proxy measure of malaria transmission, serological responses to *P*. *falciparum* antigens have shown a robust and consistent correlation with estimates of entomological inoculation rate (EIR)^10^, and thus have increasingly been incorporated in cross-sectional and longitudinal studies to monitor changes in transmission^11–15^ and identify hotspots in transmission^16, 17^.

Whilst several sero-epidemiological studies have been conducted in the low-transmission western highlands of Kenya^18–20^, no such study has been carried out in the adjacent Lake Victoria basin where prevalence is moderate to high with significant local heterogeneity^21^. In the present study, antibody responses to *P*. *falciparum* blood-stages antigens apical membrane antigen 1 (AMA-1), merozoite surface antigen-1_19_ (MSP-1_19_) and circumsporozoite antigen (CSP) were measured to assess malaria exposure and transmission on islands in Lake Victoria. Results from this study provide baseline data to evaluate the planned malaria elimination programme in the study area.

## Results

### Characteristics and parasite rates of the study participants

A total of 5044 participants were enrolled from five different settings (336–1947 individuals per site) in January and August 2012. Population coverage varied among settings: 10.5% in Mfangano, 35.7% in Ungoye and 48–90.6% in the small islands. Gender and age distributions were similar across the five settings. The majority of participants were children and adolescents ≤15 years old (73.0%, 95% CI: 71.7–74.2) and came from the islands (75.4%, 95% CI: 74.2–76.6). At enrolment, 5.9% (95% CI: 5.2–6.5) of the population were febrile (axillary temperature >37.5 °C), and 20.8% (95% CI: 19.7–22.0) were anaemic (haemoglobin [Hb] level < 11 g/dL). Of all children 12 years and below (n = 3045), 1261 (41.4%; 95% CI: 39.7–43.2) were found to have an enlarged spleen. The prevalence of febrile illness, anaemia, and enlarged spleen varied significantly by study sites (P < 0.001). Further details on the study population are shown in Table 1.

**Table 1:** Demographic characteristics of all surveyed population.

Characteristic		Study site				
	Overall	Ungoye	Mfangano	Takawiri	Kibuogi	Ngodhe
Total number of study participants	5044	1239	1947	888	336	634
Female gender, n (%)	2623 (52.2)	628 (50.7)	998 (51.3)	468 (52.9)	194 (57.7)	344 (54.3)
Age, median (IQR), years	10 (6–18)	10 (5–13)	11 (6–18)	10 (5–20)	10 (5–20)	12 (7–23)
0–5	1211 (24.0)	345 (27.9)	396 (23.4)	233 (26.3)	102 (30.4)	135 (21.3)
6–10	1319 (26.2)	362 (29.2)	526 (27.0)	228 (25.8)	72 (21.4)	131 (20.7)
11–15	1146 (22.8)	298 (24.1)	502 (25.8)	171 (19.3)	41 (12.2)	134 (21.2)
16–30	756 (15.0)	123 (9.9)	266 (13.7)	160 (18.1)	84 (25.0)	123 (19.4)
>30	606 (12.0)	110 (8.9)	256 (13.2)	93 (10.5)	37 (11.0)	110 (17.4)
Axillary temperature, Mean ± SD, °C	36.9 ± 0.6	36.9 ± 0.7	36.9 ± 0.6	36.8 ± 0.5	36.7 ± 0.5	36.8 ± 0.6
Temperature > 37.5 °C at time of survey, n (%)	295 (5.9)	118 (9.5)	105 (5.4)	32 (3.6)	9 (2.7)	31 (4.9)
Haemoglobin (Hb) level, Mean ± SD, g/dL	12.5 ± 2.1	12.5 ± 1.8	12.6 ± 2.0	12.1 ± 2.3	12.7 ± 2.1	12.2 ± 2.2
Proportion anaemic (<11 g/dL), n (%)	1049 (20.8)	214 (17.3)	368 (18.9)	245 (27.7)	66 (19.7)	156 (24.6)
Enlarged spleen, n (%)	1261 (41.4)	584/948 (61.6)	438/1160 (37.8)	129/548 (23.5)	73/180 (40.6)	37/209 (17.7)

*No gender and age recorded, n = 6; Body temperature, N = 5038; Hb level, N = 5035; Spleen test, N = 3045; IQR, interquartile range, SD; standard deviation.

**Figure 1:**
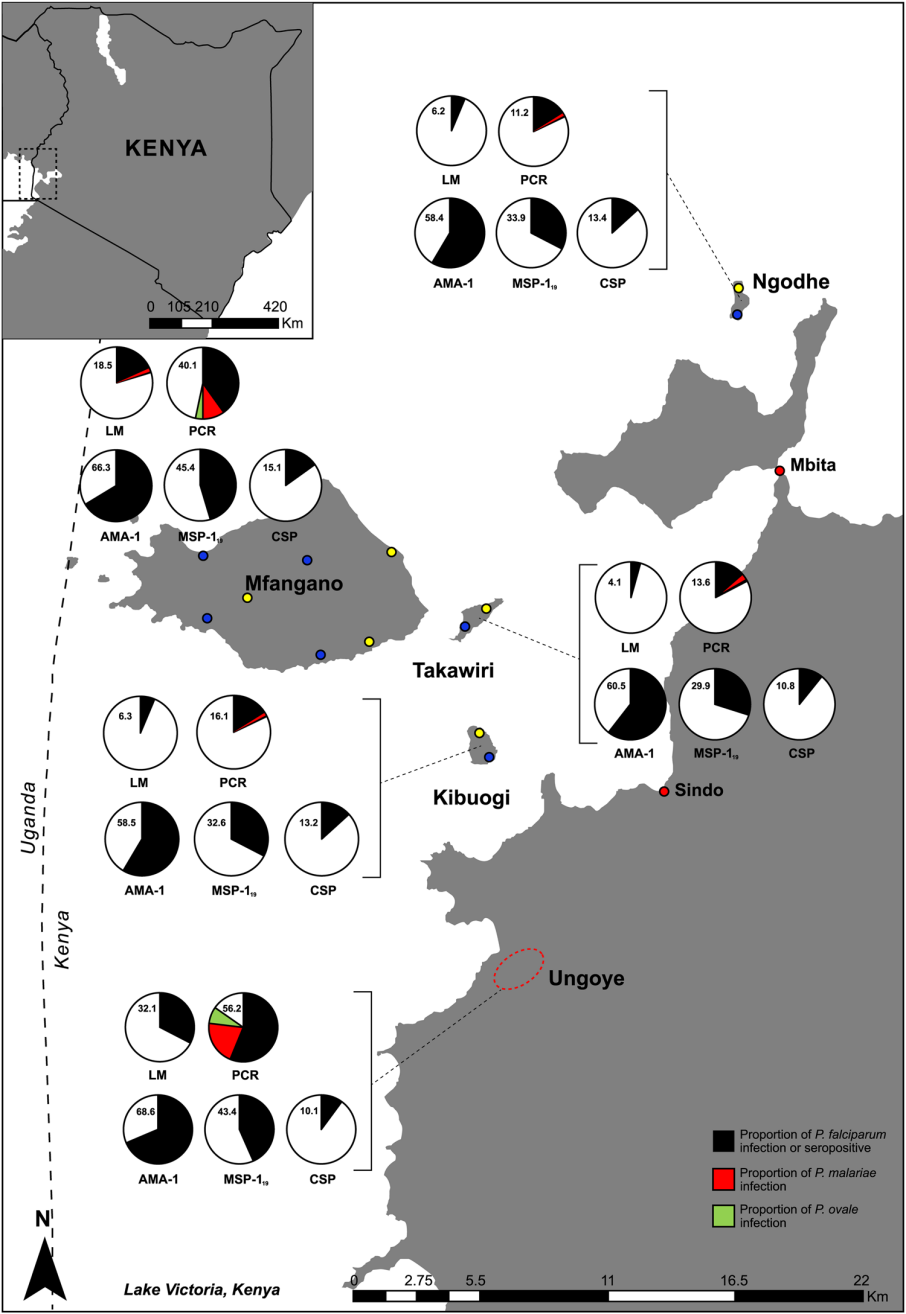
Map of the study area in Lake Victoria in western Kenya (inset) showing the proportion of *Plasmodium* spp. infection and *P*. *falciparum* seroprevalence. The population of three main areas were subjected in this study: mainland coastal village (Ungoye; area shown in red dashed line), large island (Mfangano) and three small islands (Takawiri, Kibuogi and Ngodhe). The black, red and green pies are proportions of *P*. *falciparum*, *P*. *malariae* and *P*. *ovale*, respectively. A number in each pie graph is the proportion of *P*. *falciparum* infection or seropositive. Yellow and blue circles pointed the surveyed catchment areas in January 2012 and August 2012, respectively. The most populated small towns are shown in red circle. LM is light microscopy and PCR is nested PCR. The map was created with ArcGIS software, version 10.4, http://www.esri.com.

**Figure 2:**
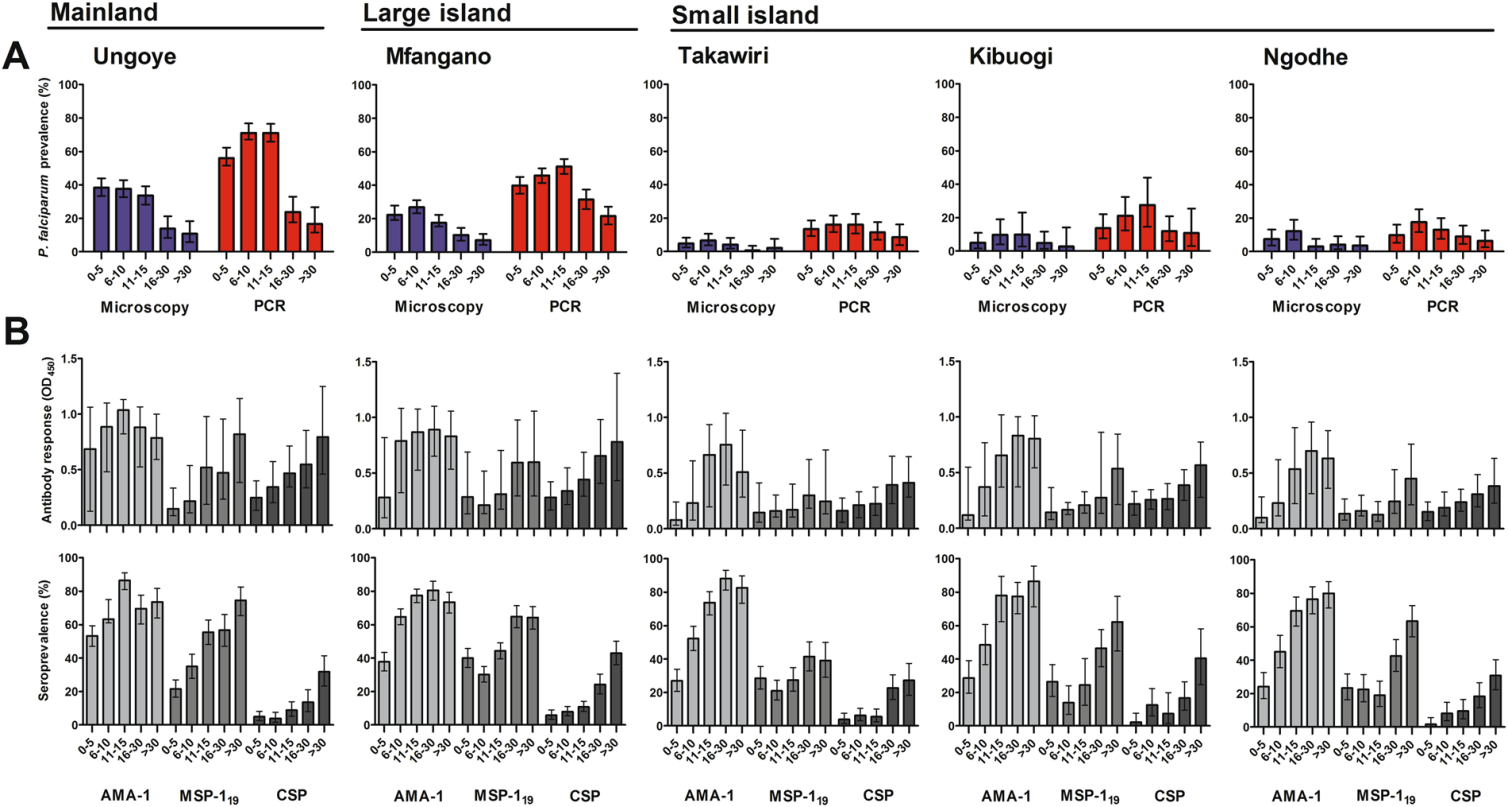
Age-specific prevalence of *P*. *falciparum* parasites and antimalarial antibody responses by setting. (**A**) The prevalence of *P*. *falciparum* (as determined by microscopy and PCR), and (**B**) optical density (OD) and respective seroprevalence to AMA-1, MSP-1_19_ and CSP (as determined by ELISA) in each setting by age group. Data are shown for both surveys combined. The total numbers of sample tested were; microscopy, n = 5012; PCR, n = 4946; ELISA, n = 4112. Error bar represents 95% confidence interval (CI) or interquartile range (IQR, 25th – 75th percentile).

The prevalence of *P*. *falciparum* infection by microscopy and PCR in the study sites is shown in Fig. 1. *P*. *falciparum* parasite prevalence ranged between 4.1 and 32.1% by microscopy, and between 11.2 and 56.2% by PCR. Parasite prevalence was significantly higher in Ungoye than other sites, regardless of detection method (P < 0.001 for all comparisons). Parasite prevalence by PCR generally peaked in the 11–15 years group and declined thereafter in all study sites (Fig. 2A). There were no statistically significant differences in mean parasite density among study sites after adjusting for age (P = 0.091). Geographic heterogeneities in malaria prevalence, sub-microscopic infections, and distribution of *Plasmodium* spp. in the study area have been reported previously^21^.

### Naturally acquired antibody responses against malaria antigens

#### AMA-1 antibodies

**Figure 3:**
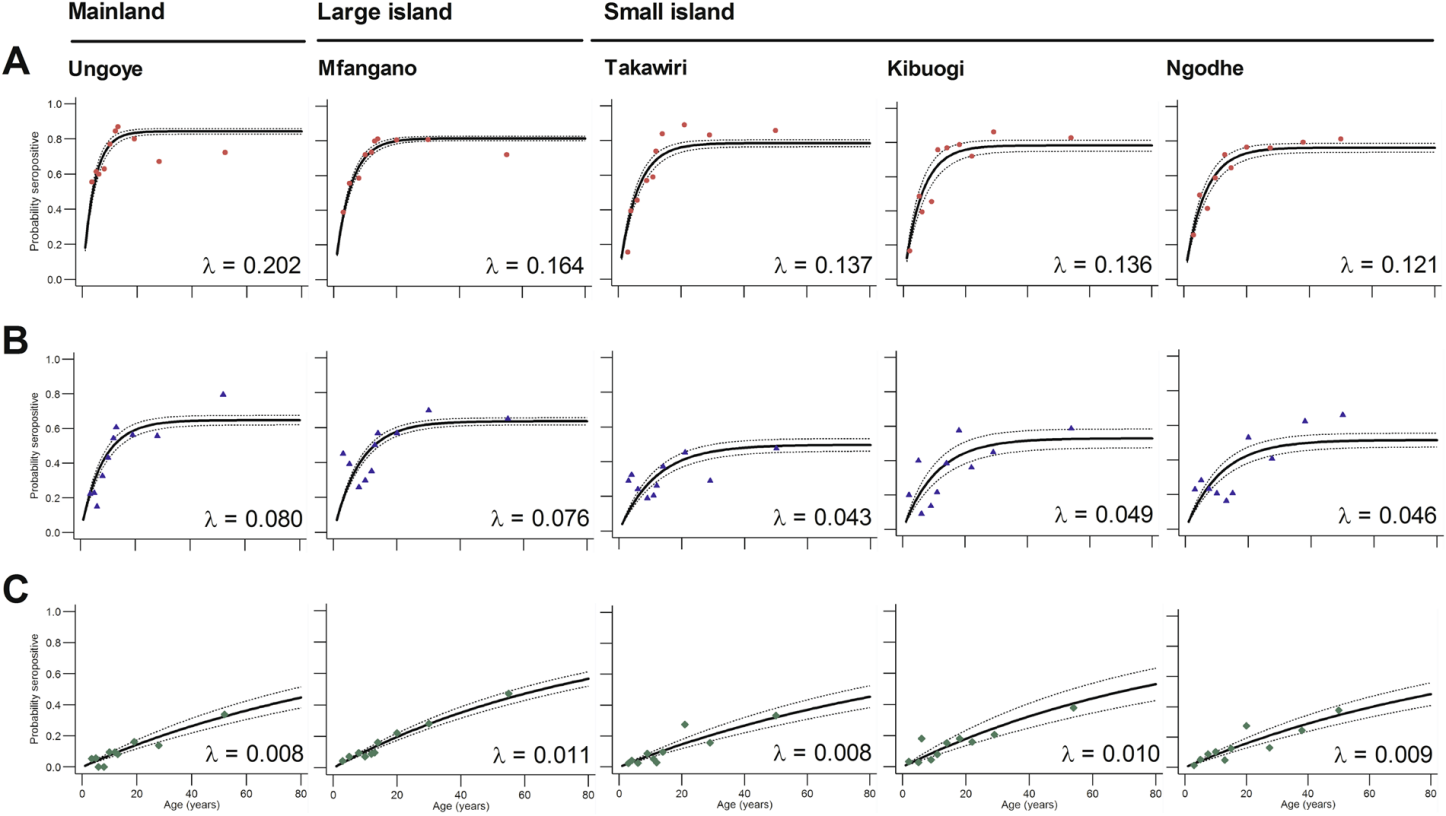
Annual probability of seroconversion rate for specific malaria antigen by age in each setting. Maximum-likelihood fits from reversible catalytic equilibrium model from each setting are shown. λ, the area-specific annual rate of seroconversion. (**A**) AMA-1, (**B**) MSP-1_19_, (**C**) CSP. The model was constrained to fit a single value for the annual probability of a common seroreversion rate (ρ), which was estimated; AMA-1: 0.0377 (95% CI 0.0312–0.0456); MSP-1_19_: 0.0433 (95% CI 0.0330–0.0568); CSP: 0.0011 (95% CI 0.0000–0.61138). Points indicated observed seroprevalence and solid lines show model-predicted seroprevalence. Broken lines are 95% confidence intervals.

**Figure 4:**
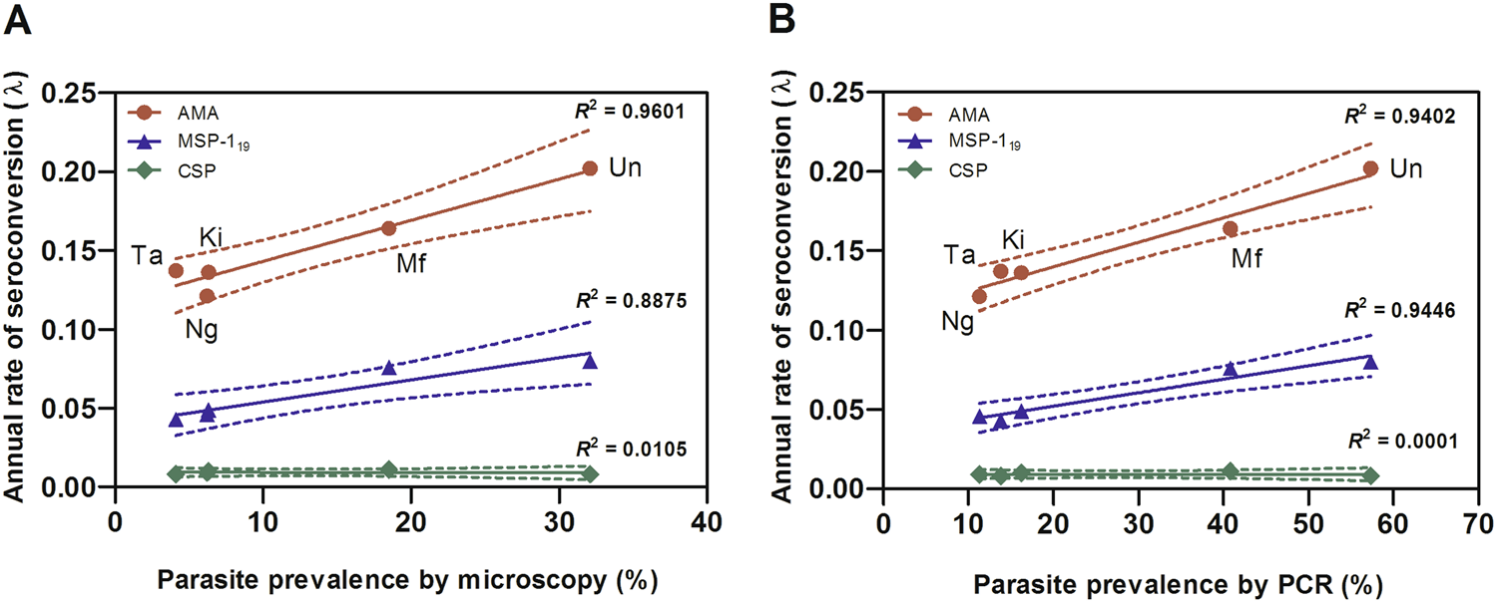
Association between the annual rate of seroconversion and *P*. *falciparum* parasite rate in the study area. Plot of estimate of seroconversion rates (λ) (calculated as for Table 2) against *P*. *falciparum* parasite rates by (**A**) microscopy, and (**B**) PCR. Un = Ungoye, Mf = Mfangano Island, Ta = Takawiri Island, Ki = Kibuogi Island, and Ng = Ngodhe Island.

Antibody responses to AMA-1, whether evaluated as OD level, antibody titre or SCR, generally increased with increasing malaria prevalence (Table 2). The overall seroprevalence was 64% (95% CI: 62.5–65.5). Seroprevalence was significantly higher (P < 0.001 for all comparisons) in Ungoye (68.6%; 95% CI: 65.3–71.6) and Mfangano (66.3%; 95% CI: 63.9–68.7) than Takawiri (60.5%; 95% CI: 56.0–64.0), Kibuogi (58.5%; 95% CI: 52.9–63.9) and Ngodhe (58.4%; 95% CI: 54.3–62.5) (Fig. 1). Seroprevalence of males and females in all study areas were comparable. Antibody responses increased with age (P < 0.001) and antibody prevalence rapidly rose in children aged 6–10 years in all study areas (Fig. 2B). More than 75% of participants became seropositive by 20 years of age (Fig. 2A), indicating rapid acquisition of antibodies with repeated infection. Estimated EIRs based on SCRs^10^ were 12, 7, 4, 4, and 3 infectious bites per person per year (ib/p/yr) for Ungoye, Mfangano, Takawiri, Kibuogi, and Ngodhe, respectively (Table 2). The age-seroprevalence curves did not indicate more than one force of infection over time; this was checked by allowing the SCR to differ at a single time point (Fig. 3A). Across the study sites, SCRs correlated significantly with *P*. *falciparum* parasite rates by microscopy (Correlation of determination, *R* ^2^: 0.9402, P = 0.009) and PCR (*R* ^2^: 0.9601, P < 0.001) (Fig. 4).

**Table 2:** Estimates of current malaria infection and exposure.

Category	Characteristic	Study site				
		Ungoye	Mfangano	Takawiri	Kibuogi	Ngodhe
Sample size	Number of sample for microscopy	1238	1938	883	336	633
	Number of sample for PCR	1237	1938	883	336	632
	Number of sample for ELISA	868	1574	770	325	575
Blood smear microscopy	Positive for *Plasmodium* spp., n (%)	403 (32.6)	393 (20.3)	38 (4.3)	21 (6.3)	39 (6.2)
	Geometric mean parasite density, parasite/µl (95% CI)	1101 (942–1286)	1045 (877–1245)	1458 (841–2526)	1665 (641–4326)	1229 (657–2298)
	*P*. *falciparum* parasite rate in children 2–10 years^1^, n/N^2^ (%)	263/669 (39.3)	211/846 (24.9)	24/419 (5.7)	11/146 (7.5)	25/234 (10.7)
	*P*. *falciparum* gametocytes rate, n/N^3^ (%)	41/397 (10.3)	34/358 (9.5)	2/36 (5.6)	5/21 (23.8)	3/39 (7.7)
PCR	Positive for *Plasmodium* spp., n (%)	1048 (84.7)	1033 (53.3)	153 (17.3)	60 (17.9)	82 (13.0)
	*P*. *falciparum* parasite rate in children 2–10 years, n/N^2^ (%)	433/669 (64.7)	366/847 (43.2)	62/420 (14.8)	26/146 (17.8)	35/234 (15.0)
ELISA	Median AMA-1 OD (IQR)	0.89 (0.48–1.08)	0.79 (0.33–1.06)	0.35 (0.09–0.82)	0.48 (0.14–0.92)	0.40 (0.13–0.83)
	Median AMA-1 antibody titre (IQR)	1579 (358–5564)	1001 (189–4083)	194 (40–1082)	295 (44–1418)	225 (47–1025)
	AMA-1 SCR, (95% CI)	0.204 (0.181–0.228)	0.166 (0.153–0.181)	0.139 (0.123–0.156)	0.136 (0.113–0.165)	0.122 (0.106–0.141)
	AMA-1 estimate EIR^4^ (95% CI)	12.1 (8.9–16.2)	7.1 (5.7–8.9)	4.4 (3.2–6.0)	4.2 (2.6–7.0)	3.2 (2.2–4.6)
	Median MSP-1_19_ OD (IQR)	0.30 (0.13–0.81)	0.33 (0.17–0.79)	0.19 (0.11–0.43)	0.20 (0.12–0.51)	0.18 (0.11–0.43)
	Median MSP-1_19_ antibody titre (IQR)	96 (33–580)	118 (45–530)	43 (24–162)	52 (28–217)	41 (22–131)
	MSP-1_19_ SCR, (95% CI)	0.079 (0.070–0.089)	0.075 (0.069–0.082)	0.043 (0.037–0.049)	0.048 (0.039–0.060)	0.046 (0.039–0.054)
	MSP-1_19_ estimate EIR^4^ (95% CI)	7.1 (5.5–9.2)	6.4 (5.4–7.7)	2.0 (1.4–2.6)	2.5 (1.6–4.0)	2.3 (1.6–3.2)
	Median CSP OD (IQR)	0.40 (0.22–0.68)	0.41 (0.26–0.72)	0.24 (0.12–0.41)	0.29 (0.20–0.44)	0.24 (0.14–0.40)
	Median CSP antibody titre (IQR)	385 (207–834)	446 (246–946)	221 (106–406)	245 (153–433)	219 (123–422)
	CSP SCR, (95% CI)	0.008 (0.006–0.010)	0.011 (0.010–0.013)	0.008 (0.006–0.010)	0.010 (0.007–0.014)	0.009 (0.007–0.011)

^1^Total of two children has no data on microscopy. ^2^Total numbers of children 2–10 years old tested. ^3^Total *P*. *falciparum* positive by slide microscopy. ^4^EIR was estimated from the SCR data using a previously described relationship^10^. OD, optical density; CI, confidence interval; IQR, interquartile range (25th–75th percentile); SCR, seroconversion rate.

#### MSP-1_19_ antibodies

Median MSP-1_19_ antibody OD levels and titres, as well as seroprevalence were lower than their corresponding AMA-1 measures across the five study sites (Fig. 1, Table 2). The overall seroprevalence was 39.5% (95% CI: 37.9–41.0). Seropositivity to MSP-1_19_ was similar (P = 0.382) between Ungoye (43.4%) and Mfangano (45.4%), and significantly lower on the three small islands (P < 0.001 for all comparisons). Seroprevalence between genders showed a higher prevalence in females in Ungoye (P = 0.023) and Mfangano (P = 0.024), but the differences were comparable in the small islands. Antibody level and seroprevalence increased significantly with age (P < 0.001) in all study areas (Fig. 2B). The acquisition of antibodies to MSP-1_19_ was moderate, with only 40% of participants becoming seropositive by 20 years of age (Fig. 3B). MSP-1_19_ SCRs were approximately 0.08 for Ungoye and Mfangano and 0.05 for the small islands, which corresponded to calculated aEIRs of 7 ib/p/yr in Ungoye and Mfangano, but only fewer than 3 ib/p/yr on the small islands (Fig. 3B, Table 2). Nevertheless, SCRs were not statistically significant across the study sites as evidenced by the overlapping confidence intervals. Similar to AMA-1, MSP-1_19_ SCRs showed significant correlation with *P*. *falciparum* parasite rates (microscopy, *R* ^2^: 0.8875, P = 0.017; PCR, *R* ^2^: 0.9446, P = 0.007) (Fig. 4).

#### CSP antibodies

The overall seroprevalence for CSP was 12.9% (95% CI: 11.9–13.9). The different serological outcomes for CSP all produced similar trends; similar between the study areas, albeit the differences in antibody responses were statistically lower than AMA-1 and MSP-1_19_ (P < 0.001 for all comparisons) (Fig. 1, Table 2). Seroprevalence of males and females in all study areas were comparable. Antibody level and seroprevalence increased with age group in all study sites (P < 0.001) (Fig. 2B), and the acquisition of antibodies to CSP was slow, with <20% of participants becoming seropositive by 20 years of age (Fig. 3C). SCRs were not significantly different among study sites and were not significantly correlated with *P*. *falciparum* parasite rate (microscopy, P = 0.869; PCR, P = 0.986) (Fig. 4A).

#### Antibody responses among *P*. *falciparum*-infected populations

**Figure 5:**
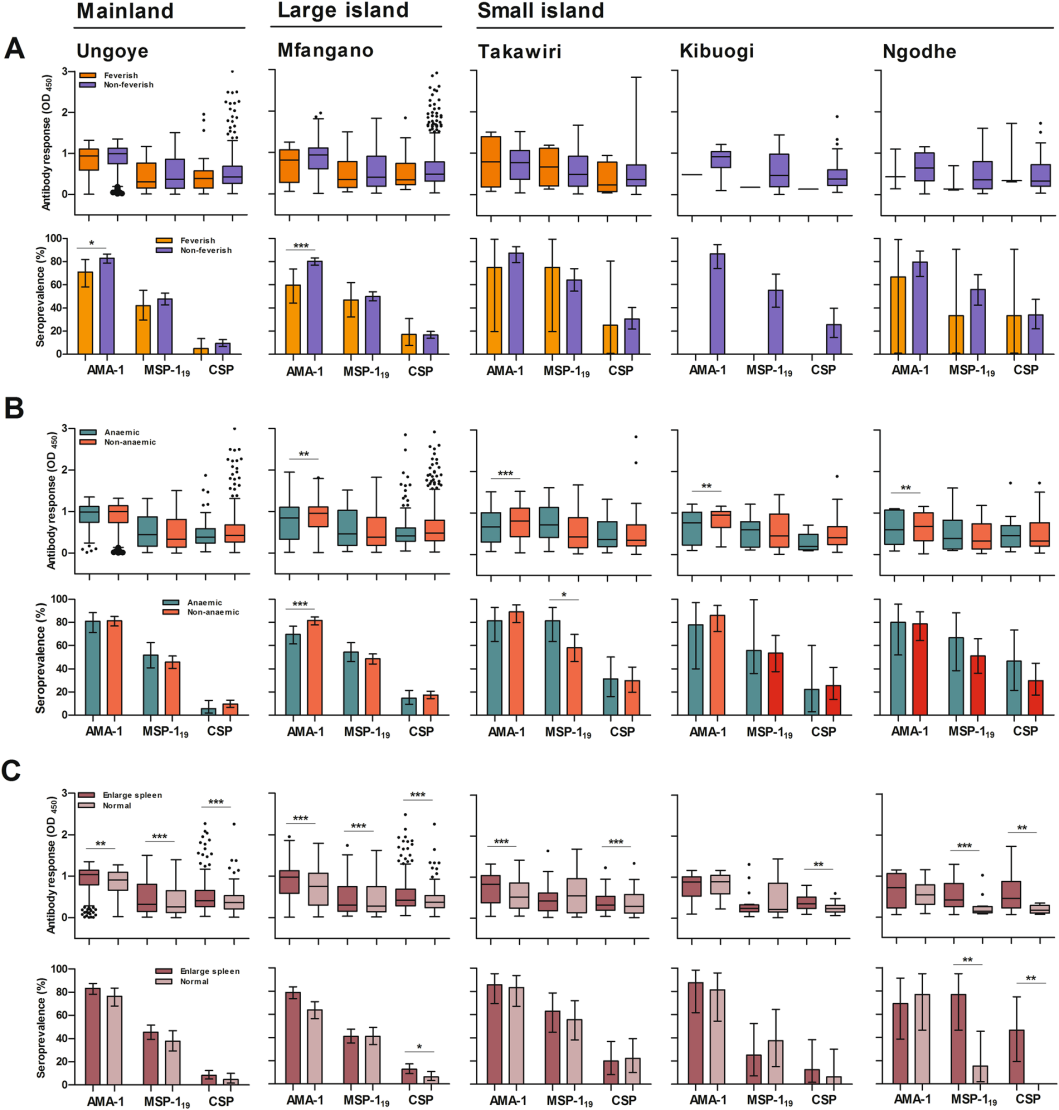
Age-adjusted antibody responses among *P*. *falciparum* infected individual (positive by PCR) for AMA-1, MSP-1_19_ and CSP in each setting. (**A**) Presented with or without fever; stratified into non-febrile (axillary temperature ≤37.5 °C) and febrile (>37.5 °C) groups. (**B**) Presented with or without anaemia; stratified into anaemic (Hb level <11 g/dL) and non-anaemic (Hb level ≥11 g/dL) groups. (**C**) Presented with or without enlarged spleen among children ≤12 years old by Hackett’s method; stratified into enlarged spleen and normal groups. Data are presented in box plots (with the median shown as a line within the box and interquartile value (IQR, 25th–75th percentile) at the edge of box) and bar graphs (with Error bar represents 95% confidence interval (CI)). Any outlier values exceeding the interquartile range are shown as circles. Differences between the two groups of antibody levels were analysed by linear regression adjusting values for age (antibody level) and Chi-square test (proportion). *, ** and *** indicates significance at P < 0.05, P < 0.01 and P < 0.001, respectively.

Figure 5 shows age-adjusted antibody responses to parasite antigens among PCR-confirmed *P*. *falciparum* infected individuals. Antibody responses did not differ significantly between the febrile and the non-febrile groups in all study sites, but the proportions of AMA-1 seropositive individuals were significantly higher (P < 0.05) in the non-febrile groups in Ungoye and Mfangano (Fig. 5A). When stratified by anaemia (Hb level < 11 g/dL), total anti-AMA-1 IgG levels were significantly higher (P < 0.01) among the non-anaemic group than the anaemic group in all island settings (Fig. 5B). Among children ≤12 years old, those with enlarged spleen had significantly higher (P < 0.01) levels of IgG against CSP in all settings. Levels of IgG against AMA-1 and MSP1_19_ were significantly higher (P < 0.01) among children with enlarged spleen than those with non-palpable spleen in Ungoye and Mfangano, although the differences were not consistently observed on the small islands (Fig. 5C).

Antibody responses to parasite antigens were analysed in relation to parasite density. Antibody levels were not significantly different between those with microscopic infections and those with sub-microscopic (microscopy-negative but PCR-positive) infections (Supplementary Fig. S1). Among those with microscopically confirmed infections, no significant differences in antibody responses were observed between those with low (<5000 parasite/µl) and high (≥5000 parasite/µl) asexual parasite densities (Supplementary Fig. S1).

#### Factors associated with seropositivity

In the adjusted model, age was significantly (P < 0.005) associated with seropositivity to AMA-1, MSP-1_19_ and CSP in all five study sites. Anaemia was significantly associated with seronegativity to AMA-1 on Mfangano (P = 0.018), Kibuogi (P = 0.008), and Ngodhe (P = 0.037) (Table 3). When restricted to children 12 years and below, enlarged spleen was significantly associated with seropositivitiy to all three parasite antigens in Ungoye (P < 0.01), Mfangano (P < 0.001), and Takawiri (P < 0.005). Enlarged spleen was also significantly associated with seropositivity to AMA-1 (P = 0.005) on Kibuogi, and seropositivity to MSP-1_19_ (P < 0.001) and CSP (P = 0.002) on Ngodhe. Age was significantly (P < 0.001) associated with AMA-1 seropositivity in all five study sites, MSP-1_19_ seropositivity in Ungoye (P < 0.001), and CSP seropositivity on Ngodhe (P = 0.024). Significant association between anaemia and MSP-1_19_ seropositivity was observed in Ungoye (P = 0.012) and Mfangano (P = 0.03) only (Table 4).

**Table 3:** Factor associated with *P*. *falciparum* seroprevalence in four islands and a mainland village, Lake Victoria Kenya, 2012*.

Setting	Factor	AMA-1		MSP-1_19_		CSP	
		aOR (95% CI)	P value	aOR (95% CI)	P value	aOR (95% CI)	P value
Ungoye	Age	1.02 (1.01–1.03)	0.002	1.06 (1.04–1.07)	<0.001	1.05 (1.03–1.06)	<0.001
Mfangano	Age	1.02 (1.01–1.03)	<0.001	1.03 (1.02–1.04)	<0.001	1.05 (1.04–1.05)	<0.001
	Anaemia	0.72 (0.56–0.95)	0.018	—	—	—	—
Takawiri	Age	1.09 (1.07–1.11)	<0.001	1.02 (1.01–1.039	0.001	1.05 (1.04–1.06)	<0.001
Kibuogi	Age	1.07 (1.04–1.09)	<0.001	1.04 (1.02–1.06)	<0.001	1.04 (1.02–1.06)	<0.001
	Anaemia	0.43 (0.23–0.80)	0.008	—	—	—	—
Ngodhe	Age	1.06 (1.04–1.07)	<0.001	1.05 81.03–1.06)	<0.001	1.05 (1.03–1.06)	<0.001
	Anaemia	0.65 (0.43–0.97)	0.037	—	—	—	—

*aOR, adjusted odd ratio; CI, confidence interval. Estimates are adjusted (i.e. age, gender, fever and anaemia) for correlation between observations from the same setting.

**Table 4:** Factor associated with *P*. *falciparum* seroprevalence among children ≤12 years old in four islands and a mainland village, Lake Victoria Kenya, 2012*.

Setting	Factor	AMA-1		MSP-1_19_		CSP	
		aOR (95% CI)	P value	aOR (95% CI)	P value	aOR (95% CI)	P value
Ungoye	Age	1.21 (1.14–1.29)	<0.001	1.21 (1.14–1.28)	<0.001	—	—
	Enlarged spleen	4.17 (2.81–6.20)	<0.001	2.30 (1.54–3.45)	<0.001	4.52 81.54–13.22)	0.006
	Anaemia	—	—	1.88 (1.15–3.00)	0.012	—	—
Mfangano	Age	1.21 (1.15–1.27)	<0.001	—	—	—	—
	Enlarged spleen	4.05 (2.95–5.53)	<0.001	1.84 (1.38–2.45)	<0.001	3.46 (1.93–5.79)	<0.001
	Anaemia	—	—	1.47 (1.04–2.08)	0.03	—	—
Takawiri	Age	1.21 (1.13–1.29)	<0.001	—	—	—	—
	Enlarged spleen	3.46 (2.07–5.78)	<0.001	2.04 (1.26–3.32)	0.004	4.31 (1.78–10.48)	0.001
Kibuogi	Age	1.25 (1.11–1.42)	<0.001	—	—	—	—
	Enlarged spleen	2.80 (1.37–5.72)	0.005	—	—	—	—
Ngodhe	Age	1.34 (1.20–1.49)	<0.001	—	—	1.27 (1.03–1.57)	0.024
	Enlarged spleen	—	—	5.11 (2.32–11.25)	<0.001	6.46 (1.95–21.38)	0.002

*aOR, adjusted odd ratio; CI, confidence interval. Estimates are adjusted (i.e. age, gender, fever, anaemia and enlarged spleen) for correlation between observations from the same setting.

## Discussion

This study describes data on parasite prevalence and antibodies responses to AMA-1, MSP-1_19_ and CSP antigens of *P*. *falciparum* in 5,044 individuals living on a coastal area and four different islands in Lake Victoria, Kenya. The present study demonstrates a clear relation of serological outcomes for AMA-1 and MSP-1_19_ with parasite prevalence and serology-derived EIR in heterogeneity of malaria transmission in the study area. AMA-1 exhibited the highest seroprevalence of the three antigens tested; AMA-1 seroprevalence was typically two- to four-fold greater than that for MSP-1_19_ and CSP between each study area. The relationship between age and parasite prevalence was similar in all five areas, increasing with age until peaking by 11–15 years and then decreasing with age. Seroprevalence and seroconversion data showed clear increases in seroreactivity with age and independently associated with enlarged spleen in children below 12 years old.

We examined the cross-sectional data to investigate the capability of antigens tested to pick up current differences in malaria prevalence. We observed heterogeneity in age-seropositivity between the study areas. Although seroprevalence showed less marked difference in *P*. *falciparum* exposure between the study areas, SCR showed a distinct variation in transmission intensity. SCRs for *P*. *falciparum* merozoite antigens (i.e. AMA-1, MSP-1_19_) were similar between Ungoye and Mfangano, but significantly higher when compared to the three small islands (Table 2, Fig. 3). Furthermore, SCRs for both AMA-1 and MSP-1_19_ consistently increased with increasing parasite prevalence in each area (Fig. 3). These results support the previous observations on the utility of AMA-1 and MSP-1_19_ antibody prevalence and rate of antibody acquisition based on SCR as a reliable estimate of the level of transmission^9, 10, 14, 22–27^. MSP-1_19_ has been proposed suitable for a wide range of transmission intensities^9, 10^, while AMA-1 suggested for low transmission setting^9, 10, 28^. The differences between the AMA-1 and MSP-1_19_ estimates in malaria exposure or transmission may be linked to differences in seroconversion and reversion rates, and this could also explain the differences observed in our study. It is likely that seroconversion and reversion rates are different for different antigens, possibly reflecting their inherent immunogenicity, subclass dependent half-life, polymorphism etc. AMA-1 appears to be more immunogenic than MSP-1_19_^9, 10^ and anti-AMA-1 titers tend to be higher than those for MSP-1_19_^9^, suggesting that seroconversion may be faster and seroreversion may be slower for AMA-1 than for MSP-1_19_.

Despite differences in immunogenicity, both *P*. *falciparum* AMA-1 and MSP-1_19_ antibody prevalence have been previously demonstrated as useful surrogate markers for malaria transmission intensity in areas of low transmission^22, 29–31^. At low transmission intensities, SCR has high sensitivity since the longevity of the antibody response toward blood-stage antigens generates higher seroprevalence rates than equivalent parasite rates^25^. As a measure that integrates exposure over time and reflects cumuative exposure rather than single current infection (e.g. microscopy), SCR based on serology can provide a more robust picture of the malaria transmission dynamics in an endemic area. In this setting, in the area with lack evidence of infections by microscopy, serological and molecular tools enabled a more complete understanding of the ongoing transmission and allowed for an examination of risk factors. Furthermore, as a metric of malaria transmission intensity, EIR is notoriously difficult to measure and the heterogeneous distribution of mosquito bites among individuals within a population makes it even more difficult to standardize^32^. Previously developed log-log calibration curves that relate SCRs to EIR values can provide a straightforward low-cost measure of malaria transmission^9, 10^. These serology-based EIR estimates, especially those based on MSP-1_19_ SCR, have shown a robust correlation with measured EIR^10^, and thus allow for clear differentiation of low-, moderate-, and high-transmission areas.

Antibody responses to CSP did not follow the same pattern as those for merozoite antigens; neither seroprevalence nor SCR showed significant difference among settings. CSP has been previously reported to give reliable estimates of malaria endemicity in hyper-endemic areas and reflect the seasonal dynamics of transmission^33–36^, making it a suitable marker for monitoring historical changes in transmission intensity^14^. AMA-1 and MSP-1_19_ are erythrocytic-stage antigens; they are produced continuously and in relative abundance because the erythrocytic-stage parasites go through several cycles of multiplication. In contrast, CSP is a pre-erythrocytic-stage antigen and is available to the immune system when a small number of sporozoites shed this antigen into circulation^37^. In consequence, the availability of CSP to immune system is of much shorter duration, which may explain its inability to detect subtle differences in exposure and transmission among settings in this study.

Factors associated to seroreactivity were identified by multivariable analyses in each study area. In all five areas, *P*. *falciparum* antibody prevalence increased with age. When analysed as a whole population, only antibody responses to AMA-1 were negatively associated with anaemia in Mfangano, Takawiri and Ngodhe. In contrast, when analysis was restricted in children ≤12 years, antibody responses to MSP-1_19_ were significantly associated with anaemia in Ungoye and Mfangano, whilst all the antigens tested were independently associated with enlarged spleen in all areas. Associations between measured antibody responses to *P*. *falciparum* antigens and the risk of malaria have shown to be inconsistent in multiple studies^38^. Potential reasons for these inconsistencies include differences in the intensity and stability of transmission, allelic variation of specific antigens and IgG subclass switching^39^, which may explain some of the differences in findings in our study areas. Nevertheless, antibodies to AMA-1 have been associated with protection from clinical malaria in some studies^38, 40, 41^, but not others^42, 43^. Some studies have also documented independent association between antibodies to MSP-1_19_ and protection from clinical malaria^42, 44^, whilst others have not^45^. Similar to the responses to blood-stage antigens, antibodies to CSP was associated with a higher odds of having an enlarged spleen, and age-adjusted antibody levels showed apparent high responses in children with enlarged spleen in each study area (Fig. 5C). This lack of protection to clinical malaria is consistent with prior studies^44, 46^ but in contrast with others^47^.

The influence of microscopically detectable parasite densities to prevalence and density of antibody responses in Kenyan children have been reported previously^48, 49^. This was only apparent in children <5 years of age and suggests that immune responses are less stable in this age group, fluctuating with concurrent infections^48, 50^. In older age groups, immune responses were not influenced by concurrent parasitaemia^35^. Contrary to previous findings, we did not find any evidence of increase antibody levels with parasite densities. Similarly, no evidence was found for a boosting of immune responses by sub-microscopic parasite carriage in our study areas, which is in agreement with a previous study in an intense malaria transmission setting in Uganda^35^, but not with other studies from low transmission settings^51, 52^. Nevertheless, sub-microscopic infections will provide an antigenic stimulus to maintain immune responses, but the role of sub-microscopic infections in maintaining immunity is yet to be quantified^53^.

This study has several limitations. First, the convenience sampling method used in this study has inherent selection bias. Most surveys were conducted in schools, meaning that children of school age (i.e. 6–15 years) were disproportionally represented (Table 1). Although this approach is valid in obtaining an estimate of antimalarial antibody prevalence^25^, over-representation of this age group among our samples likely overestimated the true malaria prevalence in the study area. Second, our surveys undersampled adult males that could have led to an underestimation of overall seroprevalence. Many adult males in the study area are engaged in fishing activities during daytime hours when our surveys were held. Malaria infections and immune status in this mobile and hard-to-reach group are not well characterised and warrant further investigation. Third, the survey design did not permit georeferencing of individual household locations and complete climate data from each study setting were unavailable. Therefore, no spatiotemporal analysis was possible to investigate patterns within individual sites or settings. A recent study screened over 800 *Plasmodium* antigens has identified serological markers of recent malaria infection^54^. Some of these markers may perform considerably better for the purpose of monitoring changes in transmission intensity.

Together these observations collectively suggest that serological analysis could be effective as an adjunct tool used in combination with parasite prevalence for surveillance, control and elimination of malaria in high endemic area. This study has demonstrated the potential of malaria antigens, namely AMA-1 and MSP-1_19_, as important markers for assessing differences in malaria transmission in a moderate to high endemic area. The concurrent measurement of parasite prevalence and serology enabled us to compare malariometric indices across different endemicity settings and confirm the heterogeneity in malaria transmission between a relatively restricted geographical areas^21^. The use of serology in assessing naturally acquired immune responses to malaria can provide promising information for future research, particularly in evaluating control interventions for malaria elimination in high transmission settings where reductions are most difficult to achieve and sustain.

## Methods

### Study area

The present study was conducted on four islands in Lake Victoria (Mfangano, Takawiri, Kibuogi, Ngodhe) and one mainland lakeshore area (Ungoye) in Homa Bay County, western Kenya (Fig. 1). Homa Bay County has an area of 4,267 square kilometres (km^2^). The population in the study area consists mainly of people of the Luo ethnic group with Dholuo is primarily spoken, as well as the national language of Kiswahili. Population estimates were 18,600 for Mfangano, 1000 for Takawiri, 700 each for Kibuogi and Ngodhe (Nagasaki University-Mbita Health and Demographic Surveillance System), and 3,471 for Ungoye (2009 Kenya Population and Housing Census). The area is predominantly rural with most residents depend on fishing and traditional small-scale farming as the main occupations^21^. Most houses are typically made of mud walls with thatched or corrugated iron roofs.

The climate in Lake Victoria is tropical, with temperatures ranging from a mean annual minimum of 17.7 °C to a mean annual maximum of 34.8 °C, and humidity is relatively high. This region generally experiences a bimodal pattern of rainfall, with the longer rainy season starting in March and ending in July and the shorter rainy season from November to December. In Lake Victoria basin, peak malaria transmission occurs 1–2 months after the rainy season and the mean monthly anopheline vector abundance has been reported to increase by 6- to 8-fold in the rainy season compared to the dry season^55^. Malaria in the study area is meso-endemic but geographically heterogeneous, with prevalence estimates ranging from 14.6% on islands to 44.2% in the coastal mainland^21^. The major vector responsible for malaria transmission in Lake Victoria is *Anopheles gambiae* s.l.^56^, and estimated annual EIR from serological markers were reported as high as 50 infectious bites per person per year^14^. *P*. *falciparum* is the predominant malaria parasite, while *Plasmodium malariae* and *Plasmodium ovale* are less common, and no *Plasmodium vivax* infections are observed^21^.

In this study, seven catchment areas on Mfangano (i.e. large island) were selected to represent the different local environments found on the island. Gulwe and Kagungu are situated in the central highland. On the other hand, Ramba, Wakinga, Mrongo, Ugina and Wakula are situated along the coastal lowlands of the island. Across the island, there are six government health facilities including one health centre and five dispensaries. Meanwhile, on each small island, population is mainly distributed between two main settlements: Kamarach and Kongata on Takawiri Island, Kibuogi A and Kibuogi B on Kibuogi Island, and Bonde and Luanda on Ngodhe Island. Takawiri and Kibuogi are close to Mfangano while Ngodhe is situated to the north of Rusinga Island. Takawiri and Ngodhe are each served by a dispensary, but no public health facility is available on Kibuogi. Ungoye, a small village on the mainland was included for comparative purposes due to its similarity to the islands in environmental characteristics, infrastructure, and access to the health facilities. It is served by a government dispensary. The village is connected by an unpaved road to the nearby towns of Sindo and Mbita, each with a sub-district hospital.

### Ethical consideration

This study was carried out in full accordance with all international, Kenyan and Swedish accepted guidelines as written informed consent was obtained from all participants. The Ethics Committee reviewed and approved all the consent procedures. This study was approved by the Kenyatta National Hospital/University of Nairobi-Ethics and Research Committee in Kenya (No.P7/1/2012) and the Committee on the Ethics of Human Research of Karolinska Institutet in Sweden (Dnr 201271239–31/4).

### Sample collections

Two cross-sectional surveys were performed in January and August 2012, after the short and long rainy seasons, respectively. Both surveys were conducted approximately 2 months after the rainy seasons to coincide with the periods of heaviest malaria burden. In our effort to ensure a high coverage of sampled population, each survey was conducted on different parts of the five study settings. The convenience sampling strategy was used in this study, whereby residents were asked to come to selected survey points such as community-based beach management unit (BMU) halls or school for study participation. Island and village leaders were sensitised to study by trained field workers and together provided information to community members at community meetings. Adult community members willing to participate were asked to read and provide signature on an informed consent form. In case of illiteracy, consent by thumb print was obtained in the presence of an independent literate adult witness. For children ≤12 years of age, consent was obtained from parents or guardians.

Different catchment areas were targeted in each survey (Fig. 1). On Mfangano Island, three (i.e. Gulwe, Ramba, Wakinga) and four (i.e. Kagungu, Mrongo, Ugina, Wakula) catchment areas were surveyed in January and August, respectively. Except for Wakula and Ugina where surveys were conducted in BMU halls, primary school surveys were carried out in other catchment areas on Mfangano. The intra-island epidemiology of malaria in this island has been described in detail elsewhere^21^. On each small island, surveys were conducted separately between the two main settlements: Kamarach, Kibuogi A and Bonde in January and Kongata, Kibuogi B and Luanda in August. All surveys on small islands were conducted at BMU halls, which are typically connected to marketplaces.

To ensure a balanced representation of all age groups, five age categories were defined per study area: one to five years, six to ten years, 11–15 years, 16–30 years, and >30 years. Age and gender of each participant were recorded. Axillary body temperature was determined using a digital thermometer (Terumo, New Jersey, US), and those with temperature exceeding 37.5 °C were considered febrile. Haemoglobin level was measured with the HemoCue Hb 201 Analyzer (HemoCue, Angelholm, Sweden). A measurement below 11 g/dL was classified as anaemic. Children aged 12 years and below were examined for enlarged spleen by AK according to Hackett’s method, regardless of fever or malaria status.

### Parasite detection by microscopy and PCR

A blood sample was obtained by finger prick (approximately 10 µl) for thick and thin blood smears, and two spots of blood (70 µl each) were collected on Whatman ET31 Chr filter paper (Whatman, UK). Blood spots on filter paper were air-dried and stored in plastic bags at 4 °C short term and at −20 °C for longer term. Blood smears were stained with Giemsa solution and examined for the presence of *P*. *falciparum* parasite in 100 high-power fields by experienced microscopists as described previously^21^. Parasite density was determined by counting the number of asexual parasites against 200 leukocytes and assuming that there are 8,000 leukocytes per µl of blood. DNA was extracted from a quartered blood spot (17.5 µl) using the QIAamp Blood Mini Kit (QIAGEN, Germantown, USA) according to the manufacturer’s instructions. The presence of *P*. *falciparum* DNA was assessed using a nested PCR protocol (nPCR) as described previously^57^.

### Serological assays

A 3 mm disk was punched from each dried blood spot and serum was eluted in reconstitution buffer in 0.5 ml deep well plates (Corning Costar, PA, USA) as described previously^58^. The reconstituted blood spot solution, equivalent to a 1/200 dilution of serum, was stored at 4 °C until used for antibody test.

All sera were tested for IgG antibodies by indirect quantitative enzyme-linked immunosorbent assay (ELISA) to two recombinant blood-stage *P*. *falciparum* malaria antigens namely apical membrane antigen-1 (AMA-1, 3D7) and merozoite surface protein-1 (MSP-1_19_, Wellcome genotype), as well as to the *P*. *falciparum* sporozoite antigen NANP_5_ repeat peptide circumsporozoite protein (CSP) (Alpha Diagnostic International, USA). Briefly, antigens were coated on NUNC-Immuno plates (Sigma) at concentrations of 0.5 µg/ml and 1.0 µg/ml in coating buffer (50 µl per well) for recombinant blood-stage antigens (AMA-1 and MSP-1_19_) and CSP, respectively. The plates were washed in PBS with 0.05% Tween 20 (PBS/T) and blocked using 1% (w/v) skimmed milk solution (Sigma, USA) in PBS/T for three hours. After washing, 50 µl of reconstituted blood spot solution were added in duplicate at final dilutions of 1:1000 for both MSP-1_19_ and CSP, and 1:2000 for AMA-1 and incubated overnight at 4 °C. A positive control consisting of a pool of hyper-immune sera was included in each plate. The plates were washed and 50 µl of horse-radish peroxidase (HRP)-conjugated rabbit anti-human IgG antibody (DAKO, Denmark) were added to all wells at a dilution 1:15,000 in PBS/T and incubated for three hours. After further series of washes, 100 µl of the substrate solution 3,3′,5,5′-tetramethylbenzidine (TMB) (tebu-bio laboratories, France) were added. Reactions were stopped after 15 minutes with 50 µl per well of 0.2 M H_2_SO_4_. The optical density (OD) at 450 nm was read using Multiskan Go ELISA reader (Thermo Fisher Scientific, USA).

### Data analysis

Statistical analysis was performed using STATA/SE version 13.1 (StataCorp, TX, USA) and GraphPad Prism Software version 5.03 (GraphPad Software Inc., CA, USA). Duplicate ELISA OD values were averaged and normalised against values from blank wells as previously described to adjust for background reactivity^58^. Seropositivity was determined by fitting a mixture model to normalised OD values assuming two Gaussian distributions, one for sero-negative individuals and another for sero-positive individuals^25^. The mean OD plus three standard deviations associated with the sero-negative group was used as the cut-off value for seropositivity. A separate cut-off was generated for each antigen. Differences in parasite prevalence and seroprevalence estimates between study areas and age categories were performed using a two-sided test for proportions and the corresponding exact binomial 95% confidence intervals (95% CIs). The titre of antibody responses was estimated using the equation dilution/[maximum OD/(OD test serum – minimum OD) – 1]; the median titre and interquartile range (IQR) are given. Differences in antibody responses were assessed using age-adjusted linear regression. Seroprevalence was stratified into yearly age groups and then analysed using a reverse catalytic modelling approach under a binomial sampling assumption, as described elsewhere^9, 10, 13^. This method provides estimates of the mean annual rates of conversion to seropositive (seroconversion rate, SCR [λ]) and reversion to seronegative (seroreversion rate, SRR [ρ]) status, averaged over the age of the population. The common SRR was estimated from the model using maximum likelihood as described previously^9^. Infant under 1 year of age were excluded to remove any influence of maternally derived antibodies^9^. The serologically-derived annual EIR was estimated using AMA-1 and MSP-1_19_ seroconversion rates and a calibration curve derived from determined values^10^. Factors associated with *P*. *falciparum* seropositivity were determined for each site separately using generalised estimating equations adjusting for correlation between observations from the same variables. The following factors were included: age in years, gender, fever, anaemia and enlarged spleen (children ≤12 years old). Variables that were significant at P < 0.10 in the univariate analyses were added to the multivariate model and retained in the final multivariate model if their association with immune responses was statistically significant at P < 0.05.

### Data availability

The datasets generated during and/or analysed during the current study are available from the corresponding author on reasonable request.

## Electronic supplementary material

Supplementary Figure S1
