# Supplementary material for: Naturally acquired antibody response to Plasmodium falciparum describes heterogeneity in transmission on islands in Lake Victoria
Source: Sci Rep. 2017 Aug 22;7:9123. doi: 10.1038/s41598-017-09585-4 (PMC5567232; doi:10.1038/s41598-017-09585-4)

Naturally acquired antibody response to *Plasmodium falciparum* describes heterogeneity in transmission on islands in Lake Victoria

Zulkarnain Md Idris, Chim W. Chan, James Kongere, Tom Hall, John Logedi, Jesse Gitaka, Chris Drakeley, Akira Kaneko

Supplementary Figure Legend

Supplementary Figure S1. Age-adjusted antibody responses among the different levels of *P. falciparum* positive individual for AMA-1, MSP-1_{19} and CSP in each setting. **(A)** *P. falciparum* detected by microscopy and PCR; stratified into microscopic and sub-microscopic groups. **(B)** Level of asexual parasitaemia; stratified into low (<5000 parasites/µL) and high (≥5000 parasites/µL) parasitaemia groups. Data are presented in box plots with the median shown as a line within the box and interquartile value (IQR, 25th – 75th percentile) at the edge of box. Any outlier values exceeding the interquartile range are shown as circles. Differences between the two groups were analysed by linear regression, adjusting values for age.

Supplementary Figure S1

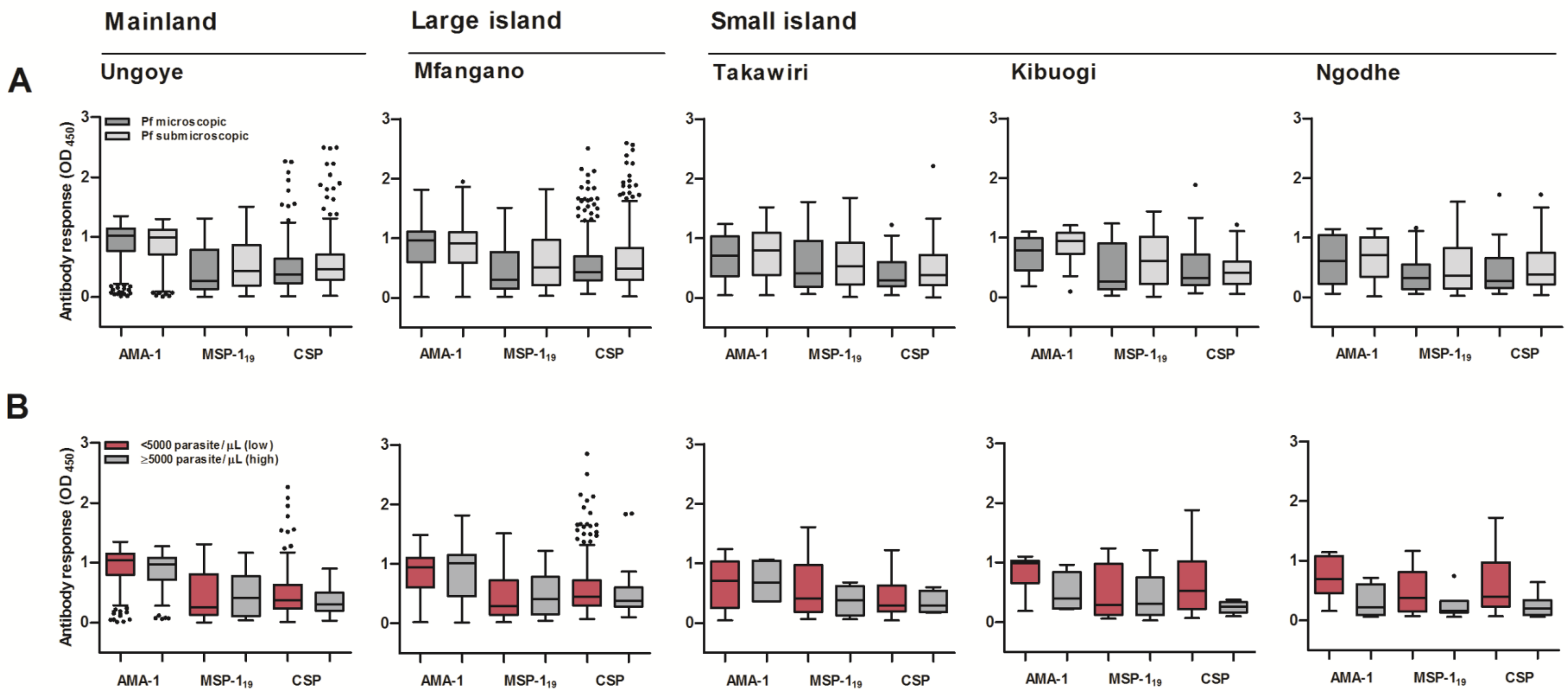

Supplement: Supplementary file 1 — Supplementary Figure S1 [file 41598_2017_9585_MOESM1_ESM.pdf]
